# Antibacterial activity of the antimicrobial peptide PMAP-36 in combination with tetracycline against porcine extraintestinal pathogenic *Escherichia coli* in vitro and in vivo

**DOI:** 10.1186/s13567-024-01295-w

**Published:** 2024-03-22

**Authors:** Qi Tao, Yi Lu, Qian Liu, Runqiu Chen, Yating Xu, Gang Li, Xiaoxiang Hu, Chao Ye, Lianci Peng, Rendong Fang

**Affiliations:** Joint International Research Laboratory of Animal Health and Animal Food Safety, College of Veterinary Medicine, Southwest University, Chongqing, 400715 China

**Keywords:** Porcine extraintestinal pathogenic *Escherichia coli*, PMAP-36, synergistic effects, multidrug resistance

## Abstract

The increase in the emergence of antimicrobial resistance has led to great challenges in controlling porcine extraintestinal pathogenic *Escherichia coli* (ExPEC) infections. Combinations of antimicrobial peptides (AMPs) and antibiotics can synergistically improve antimicrobial efficacy and reduce bacterial resistance. In this study, we investigated the antibacterial activity of porcine myeloid antimicrobial peptide 36 (PMAP-36) in combination with tetracycline against porcine ExPEC PCN033 both in vitro and in vivo. The minimum bactericidal concentrations (MBCs) of AMPs (PMAP-36 and PR-39) against the ExPEC strains PCN033 and RS218 were 10 μM and 5 μM, respectively. Results of the checkerboard assay and the time-kill assay showed that PMAP-36 and antibiotics (tetracycline and gentamicin) had synergistic bactericidal effects against PCN033. PMAP-36 and tetracycline in combination led to PCN033 cell wall shrinkage, as was shown by scanning electron microscopy. Furthermore, PMAP-36 delayed the emergence of PCN033 resistance to tetracycline by inhibiting the expression of the tetracycline resistance gene *tetB*. In a mouse model of systemic infection of PCN033, treatment with PMAP-36 combined with tetracycline significantly increased the survival rate, reduced the bacterial load and dampened the inflammatory response in mice. In addition, detection of immune cells in the peritoneal lavage fluid using flow cytometry revealed that the combination of PMAP-36 and tetracycline promoted the migration of monocytes/macrophages to the infection site. Our results suggest that AMPs in combination with antibiotics may provide more therapeutic options against multidrug-resistant porcine ExPEC.

**Supplementary Information:**

The online version contains supplementary material available at 10.1186/s13567-024-01295-w.

## Introduction

Most strains of *Escherichia coli* are non-pathogenic to humans and animals, but some strains are pathogenic, such as diarrhoeagenic and intestinal pathogenic *E. coli* (InPEC) and extraintestinal pathogenic *E. coli* (ExPEC) [1]. ExPEC has recently emerged as a fatal pathogen that causes pneumonia, septicaemia, and meningitis, resulting in considerable economic losses in the pig industry worldwide [2, 3]. Moreover, the increasing prevalence of resistance to conventional antibiotics in ExPEC has made treatment increasingly challenging [4]. Thus, there is an urgent need to develop new alternatives or additives to antibiotics to combat multidrug-resistant ExPEC.

Cathelicidins (CATHs) are short (<40 amino acid residues), cationic, antimicrobial peptides (AMPs) that have been identified in a variety of vertebrate species [5]. Porcine CATHs include PR-39, PG1-5 (protegrin), PF1-2 (prophenin), and porcine myeloid antimicrobial peptides (PMAPs) [6]. Porcine CATHs have emerged as promising antibacterial agents due to their broad-spectrum antibacterial activity against different multidrug-resistant gram-positive and gram-negative bacteria [7, 8]. CATHs not only interact with cell membranes but also regulate intracellular targets, which allows them to kill bacteria in multiple ways [9]. Therefore, bacteria are less likely to develop drug resistance to CATHs then they are to conventional antibiotics because of the specific bactericidal mechanism of CATHs. CATHs have synergistic effects with conventional antibiotics against *Clostridium difficile* (*C. difficile*), *Staphylococcus aureus* (*S. aureus*) and *Pseudomonas aeruginosa* (*P. aeruginosa*) [10, 11].

In this study, we investigated the synergistic antibacterial effects of AMPs, including PMAP-36 and PR-39 in combination with conventional antibiotics against multidrug-resistant ExPEC (PCN033) in vitro and in vivo. The results showed that porcine CATH PMAP-36 had good synergistic antibacterial activity with tetracycline. In addition, the combination of PMAP-36 with tetracycline further enhanced the anti-inflammatory effects in the host during infection and promoted the recruitment of monocytes/macrophages. In this study, we identified a new candidate antimicrobial agent.

## Materials and methods

### Bacterial strains

ExPEC PCN033 (O11:K2) [GenBank: CP006632.1] is a multidrug resistant strain isolated from the brain of a diseased swine in China [12, 13]. ExPEC RS218 (O18:K1:H7) [GenBank: CP007149.1] was isolated from the cerebrospinal fluid of a neonate with meningitis, and its final whole-genome sequence has been annotated [14]. These two strains were subsequently grown in Luria–Bertani (LB) broth or on LB agar plates at 37 °C.

### Antibiotics and peptides

All peptides (Table 1) were synthesized by China Peptides Co., Ltd. (Shanghai, China) to a purity of > 95%. All antibiotics were purchased from Macklin Biochemical Technology Co., Ltd. (Shanghai, China). The antibiotics used included streptomycin, oxacillin, cefotaxime, tetracycline, gentamicin, and ampicillin. All the agents were dissolved in distilled water and stored at −80 °C after sterilization via 0.22 μm filters.

**Table 1:** **Characteristics of the peptides used in this study**.

Peptide	Amino acid sequence	Length		Charge
CATH-1	RVKRVWPLVIRTVIAGYNLYRAIKKK		26	+ 8
CATH-2	RFGRFLRKIRRFRPKVTITIQGSARF		26	+ 9
CATH-3	RVKRFWPLVPVAINTVAAGINLYKAIRRK		29	+ 7
CATH-B1	PIRNWWIRIWEWLNGIRKRLRQRSPFYVRGHLNVTSTPQP		40	+ 7
CRAMP	GLLRKGGEKIGEKLKKIGQKIKNFFQKLVPQPEQ		34	+ 6
PMAP-36	GRFRRLRKKTRKRLKKIGKVLKWIPPIVGSIPLGCG		36	+ 13
PR-39	RRRPRPPYLPRPRPPPFFPPRLPPRIPPGFPPRFPPRFP		39	+ 10

### Cells

Primary peritoneal macrophages (PECs) were collected as previously reported [15]. Briefly, mice were intraperitoneally injected with 2 mL of 4% thioacetate (Aiken, Tokyo, Japan). After 3–4 days, mouse peritoneal exudate cells were collected by intraperitoneal lavage and suspended in RPMI 1640 (Gibco, CA, USA) supplemented with 10% foetal bovine serum (FBS). Then, the cells were seeded at a density of 2 × 10^5^ cells/well in 48-well plates and maintained in a humidified 37 °C incubator with 5% CO_2_. After 2 h of incubation, the nonadherent cells were removed, and the adherent cells were used for the assays described below.

Porcine kidney-15 (PK-15) cells (Procell Life Science & Technology Co., Ltd., Wuhan, China) were cultured at 37 °C with 5% CO_2_ in Dulbecco’s modified Eagle’s medium (Gibco, CA, USA) supplemented with 10% FBS, 100 U penicillin/mL and 100 μg streptomycin/mL. Cells were seeded at a density of 1 × 10^5^ cells/well in 48-well plates and cultured overnight before being used for the assays described below.

### In vitro antimicrobial activity assay

The minimum inhibitory concentrations (MICs) of seven antimicrobial peptides (CATH-1, -2, -3, B1, CRAMP, PMAP-36, and PR-39) and six antibiotics (streptomycin, oxacillin, cefotaxime, tetracycline, gentamicin, and ampicillin) were determined using the Mueller–Hinton broth (MHB) microdilution method recommended by the CLSI. Briefly, 50 μL of a mid-logarithmic phase bacterial suspension (2 × 10^6^ CFU/mL) was mixed with an equal volume of peptides (1.25–80 μM) and antibiotics (1.25–81,920 μM) in triplicate in MHB and incubated for 16–20 h at 37 °C prior to MIC determination. The MIC was defined as the lowest concentration of a compound that inhibited visual growth of bacteria. The minimum bactericidal concentration (MBC) was determined by colony counting. Fifty microlitres of bacterial culture was removed from wells in which visible growth was not observed and plated on LB agar plates. After overnight culture at 37 °C, the surviving bacteria were counted.

### Synergistic antibacterial activity of AMPs and antibiotics in vitro

The synergistic antibacterial activity of AMPs (PMAP-36 and PR-39) and antibiotics (tetracycline and gentamicin) against PCN033 and RS218 was determined by a conventional checkerboard assay and evaluated by the fractional bactericidal concentration index (FBCI) as reported previously [16]. In brief, 25 μL of twofold serial dilutions of AMPs (ranging from 2 MBC to 1/32 MBC) were added to vertical wells, and then 25 μL of twofold dilutions of antibiotics (ranging from 2 MBC to 1/512 MBC) were added to horizontal wells in 96-well plates. Subsequently, 50 μL of bacterial suspension (2 × 10^6^ CFU/mL) was added and the samples were incubated at 37 °C for 18 h. After incubation, the plates were visually inspected for turbidity to determine growth. Next, 50 μL of mixed medium was removed from wells in which visible growth was not observed, and the media were subsequently plated on LB agar plates. The FBCI was calculated according to the formula FBCI = (C_AMPs_/MBC_AMPs_) + (C_antibiotics_/MBC_antibiotics_), in which MBC_AMPs_ and MBC_antibiotics_ represent the MBCs of AMPs and antibiotics alone, respectively, and C_AMPs_ and C_antibiotics_ represent the concentrations of AMPs and antibiotics, respectively, when they are used in combination. The FBCIs were defined as follows: synergy was defined as FBCI < 0.5, additivity was defined as 0.5 < FBCI ≤ 1, indifference was defined as 1 < FBCI ≤ 2, and antagonism was defined as FBCI > 2.

### Time-kill curve assay

A bacterial suspension of PCN033 was prepared as described above. Fifty µL of PMAP-36 at 1/4 MBC, tetracycline at 1/16 MBC, gentamicin at 1/8 MBC, and PMAP-36 in combination with tetracycline or gentamicin solutions were prepared in 96-well plates. Then, the same volume of bacterial suspension (2 × 10^6^ CFU/mL) was added. Untreated bacteria were used as a growth control, and the cultures were incubated at 37 °C. The CFU/mL values of the cultures were determined after 0, 5, 10, 20, 30, 60, and 120 min of incubation. After incubation, 50 µL samples were taken, diluted tenfold in MHB, and then plated on LB agar plates in triplicate at 37 °C. After overnight culture, the viable colonies were counted and recorded as log_10_ CFU/mL.

### Scanning electron microscopy (SEM) analysis

PCN033 (1 × 10^8^ CFU/mL) was incubated with or without tetracycline (1/16 MBC), PMAP-36 (1/4 MBC), or tetracycline (1/16 MBC) + PMAP-36 (1/4 MBC) in 3 mL of MHB at 37 °C for 3 h. After incubation, the bacterial suspension was centrifuged and washed with sterile PBS three times. Then, the bacterial pellets were fixed with 1.5 mL of 2.5% (v/v) glutaraldehyde-PBS at 4 °C for 24 h. Finally, the samples were sent to the Lilai Biomedicine Experiment Center (Sichuan, China) for SEM analysis.

### Quantitative RT‒PCR

RT‒PCR assays were used to determine the effect of PMAP-36 on transcription of the drug resistance gene *tetB*. In brief, bacterial suspensions (1 × 10^6^ CFU/mL) were incubated with tetracycline (1/32 MIC), tetracycline (1/32 MIC) + PMAP-36 (1/32 MIC) or different concentrations of PMAP-36 in MHB at 37 °C for 6 h. After incubation, total RNA was extracted using a Bacterial RNA Extraction Kit (Omega Biotek, Norcross, USA) according to the manufacturer’s instructions. Then, the RNA was reverse transcribed to cDNA using the PrimeScript^®^ RT Reagent Kit (TaKaRa, Dalian, China) according to the manufacturer’s instructions. Subsequently, the level of *tetB* was determined by qRT‒PCR using a CFX96 Real-time PCR detection system (Bio-Rad, USA). The sequences of the primers used in this study were as follows: 16S rRNA forward 5′-TGC CTG ATG GAG GGG GAT AA and reverse 5′-CCA GTG TTG CTG GTC ATC CT; and *tetB* forward 5′-TGG CCT ATC AAT TGC GCT GA and reverse 5′-AGC GGG GCC TAT TAT TGG TG. 16S RNA was selected as the reference, and the relative mRNA expression levels of *tetB* were calculated according to the 2^*−ΔΔCt*^ method.

### Cytotoxicity and haemolytic activity assay

Cytotoxicity assays were performed in mouse peritoneal macrophages and PK-15 cells using a WST-1 assay. Cells were seeded in a 48-well plate and incubated with or without PMAP-36 (2.5 μM), tetracycline (10 μM), gentamicin (160 μM), tetracycline (10 μM) + PMAP-36 (2.5 μM), or gentamicin (160 μM) + PMAP-36 (2.5 μM) for 2 h at 37 °C with CO_2_. After 2 h of incubation, the cells were washed and cultured for 22 or 46 h. Afterwards, the medium was replaced with 150 μL of culture medium containing 10% WST-1 (Roche, Basel, Switzerland) and incubated for 20–30 min. The absorbance at 450 nm was measured using a microplate reader (Bio-Rad, Japan) with background correction at 630 nm. Cell viability was determined using the nontreated sample as 100% viability.

The haemolytic activity of PMAP-36 and antibiotics was determined using mouse erythrocytes as previously described [16]. In brief, aliquots of 100 μL of a 2% suspension of erythrocytes were mixed with 100 μL of the test compounds in PBS in 96-well plates and incubated for 1 h at 37 °C. After centrifugation at 1500 rpm for 5 min, the supernatants were collected, and the absorbance at 570 nm was determined. PBS and 1% Triton X-100 were used as negative and positive controls, respectively. Haemolysis percentage was calculated using the following formula: [(OD_570 nm_ of treated sample – OD_570 nm_ of negative control)/(OD_570 nm_ of positive control – OD_570 nm_ of negative control)] × 100%.

### In vitro resistance induction

Antibiotic resistance was induced in PCN033 as previously reported [16]. A bacterial suspension (2 × 10^6^ CFU/mL) was grown independently with tetracycline (1/4 MIC), PMAP-36 (1/4 MIC), or tetracycline (1/8 MIC) + PMAP-36 (1/8 MIC) in MHB at 37 °C overnight for 30 consecutive generations. Bacteria from the highest drug combination were regrown, the MIC of tetracycline was measured, and the bacteria were then treated with the drug combination again. The change in MIC was determined by normalizing the MIC of the n generation to the MIC of the first generation.

### Mouse infection

Female C57BL/6 mice (6–8 weeks old) were obtained from Chongqing Lepitt Biotechnology Co., Ltd. This study was approved by the Institutional Animal Care and Use Committee (IACUC) of Southwest University, Chongqing, China (IACUC-20221022-18). Mice were intraperitoneally infected with 1 × 10^7^ CFU of PCN033 in 200 µL of sterile PBS and randomly divided into four groups: (i) untreated control; (ii) 30 mg/kg tetracycline; (iii) 1 mg/kg PMAP-36; and (iv) 30 mg/kg tetracycline + 1 mg/kg PMAP-36. After 2 h of infection, the mice were intraperitoneally injected with tetracycline and PMAP-36. Afterwards, different assays were performed as described below.

For the generation of survival curves, mice (*n* = 10/group) were monitored every 6 h until 72 h post-infection. Statistical analysis was conducted by the log rank (Mantel‒Cox) test in GraphPad Prism 8.0 software.

Mice (*n* = 5/group) were euthanized at 12 h post-infection. Then, organs (spleen, liver, and lung), blood and peritoneal lavage fluid (PLF) were collected and homogenized to determine the bacterial load via serial tenfold dilutions, plating on LB agar plates, and incubation at 37 °C overnight.

For histopathological observation, mice (*n* = 3/group) were euthanized at 12 h post-infection. Then, the lung tissues were fixed with 10% formaldehyde for over one week and embedded in paraffin for histological analysis.

For cytokine detection, the mice (*n* = 3/group) were euthanized at 12 h post-infection. Then, the expression of inflammatory cytokines (IL-1α, IL-1β, IL-6, IL-12 and TNF-α) in the lungs, liver, spleen, and PLF and the expression of chemokines (CXCL1 and CXCL2) in the PLF were determined using ELISA kits. The kits for IL-1α, IL-1β, IL-6, IL-12 and TNF-α were purchased from Invitrogen (CA, USA), and the kits for CXCL1 and CXCL2 were purchased from MultiSciences (Hangzhou, China).

### Flow cytometry analysis

Mice were infected and treated with the test compounds as described above. After 12 h of infection, PLF was collected with 2 mL of sterile PBS, and the cells were centrifuged at 1800 rpm for 3 min. Then, the cells were blocked with 1 mL of 2% bovine serum albumin (BSA) in PBS for 30 min. An anti-mouse CD16/32 antibody (1:50) (BioLegend, CA, USA) was used to block nonspecific antibody binding in 0.5% BSA buffer for 30 min at 4 °C. Next, the cells were stained with PE-conjugated anti-mouse F4/80 antibody (1:50) (BioLegend, CA, USA) and FITC-conjugated anti-mouse Ly-6G/Ly-6C (Gr-1) antibody (1:200) (BioLegend, CA, USA) in 0.5% BSA for 30 min at 4 °C. Afterwards, the cells were washed twice with 150 μL of 2% BSA and analysed using flow cytometry.

### Statistical analysis

Statistical analysis was performed using GraphPad Prism 8.0 software (San Diego, CA, USA), and all the data are presented as the mean ± standard deviation (SD). Student’s *t* test was used to determine the significance of differences between two groups. Statistical significance is indicated by *p < 0.05; **p < 0.01; and ***p < 0.001.

## Results

### Antibacterial activity of AMPs and antibiotics

First, we determined the MICs and MBCs of different AMPs (CATH-1, CATH-2, CATH-3, CATH-B1, CRAMP, PMAP-36, and PR-39) and antibiotics (streptomycin, oxacillin, cefotaxime, tetracycline, gentamicin, and ampicillin) in PCN033 and RS218. As shown in Table 2. Compared to other AMPs, porcine AMPs, including PMAP-36 and PR-39, showed better antibacterial activity, with MBCs between 5 μM and 20 μM against PCN033 and RS218. Cefotaxime showed good antibacterial activity, with MICs and MBCs less than 0.625 μM. The MICs of tetracycline were between 2.5 and 320 μM, and the MBCs were between 5 and 320 μM. Streptomycin, oxacillin, gentamicin, and ampicillin showed weak antibacterial activity, with MICs greater than 160 μM and MBCs greater than 1280 μM. However, gentamicin and ampicillin showed potent antibacterial activity against RS218, with MICs between 2.5 and 10 μM and MBCs between 5 and 20 μM.

**Table 2:** **Antibacterial activity of individual AMPs and antibiotics against PCN033 and RS218**.

Antimicrobials	*E. coli* PCN033		*E. coli* RS218	
	MIC (μM)	MBC (μM)	MIC (μM)	MBC (μM)
CATH-1	10	20	5	10
CATH-2	10	20	5	10
CATH-3	10	20	5	10
CATH-B1	> 40	> 40	> 40	> 40
CRAMP	10	20	5	20
PAMP-36	2.5	10	2.5	5
PR-39	5	10	2.5	5
Streptomycin	160	40,960	40,960	> 40,960
Oxacillin	5120	10,240	320	1280
Cefotaxime	< 0.625	< 0.625	< 0.625	< 0.625
Tetracycline	160	160	2.5	5
Gentamicin	320	1280	2.5	5
Ampicillin	20,480	40,960	5	10

### Synergistic antibacterial effect of AMPs in combination with antibiotics in vitro

**Figure 1:**
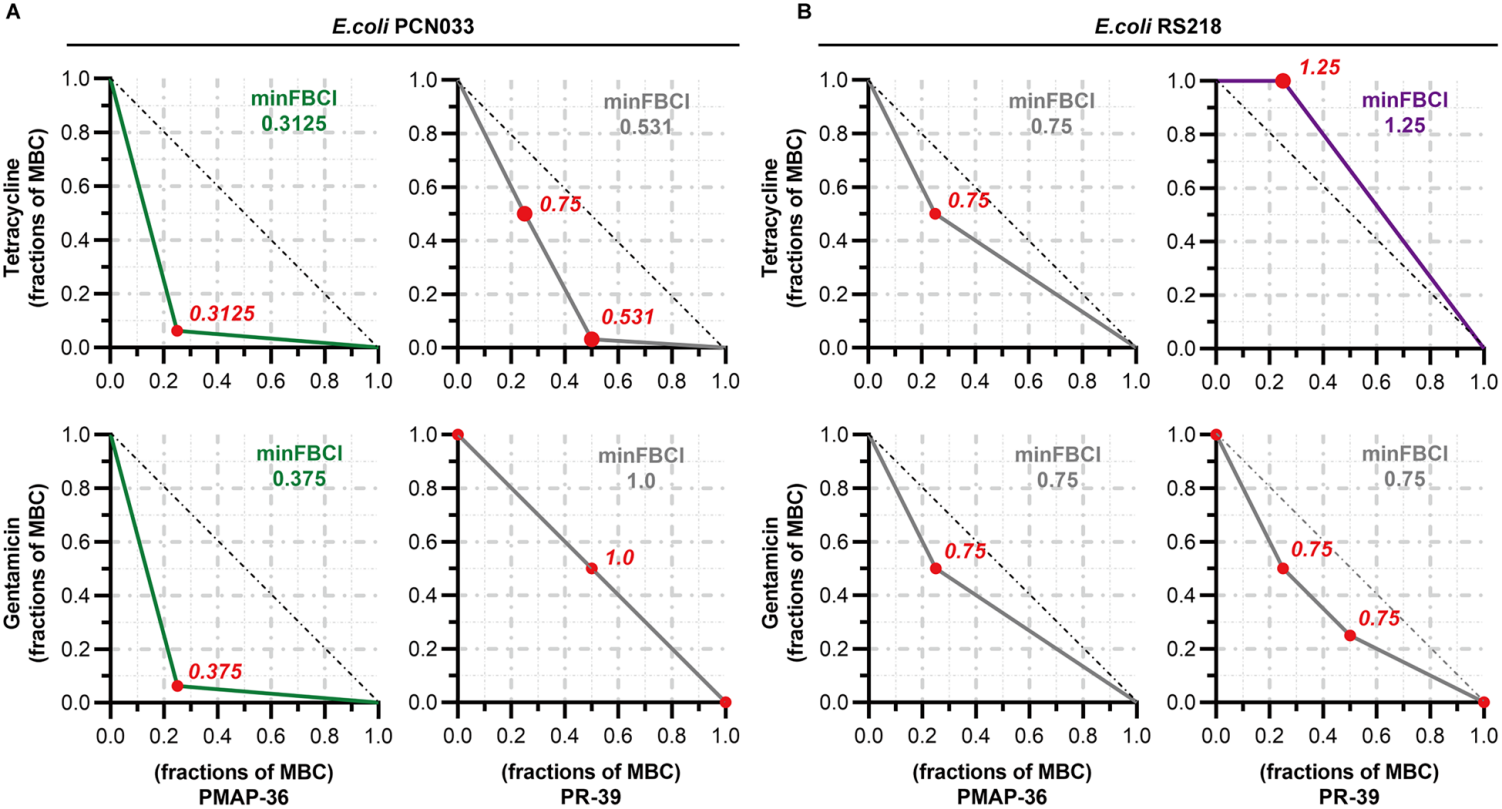
**Synergistic antibacterial effect of AMPs combined with antibiotics in vitro.** The synergistic antimicrobial activity of combinations of AMPs (PMAP-36 and PR-39) and antibiotics (tetracycline and gentamicin) against multidrug-resistant ExPEC PCN033 (**A**) and RS218 (**B**) is shown. The synergistic antibacterial activity was determined by the fractional bactericidal concentration index (FBCI). In each contour map, different coloured lines, including green, grey, and purple, represent synergy, additivity, and independence, respectively.

Checkerboard assays were performed to evaluate the synergistic antibacterial effect of PMAP-36 in combination with tetracycline or gentamicin. The results showed that PMAP-36 in combination with tetracycline or gentamicin had strong synergistic antimicrobial effects on PCN033, with FBCIs of 0.3125 and 0.375, respectively (Figure 1A). The combination of PR-39 and antibiotics (tetracycline or gentamicin) against PCN033 had an additive effect (0.5 < FBCIs ≤ 1, Figure 1A). For anti-RS218 activity, the FBCIs of these four combinations were > 0.5, indicating that they had no synergistic effect (Figure 1B).

### Antibacterial efficacy of PMAP-36 in combination with antibiotics in vitro

**Figure 2:**
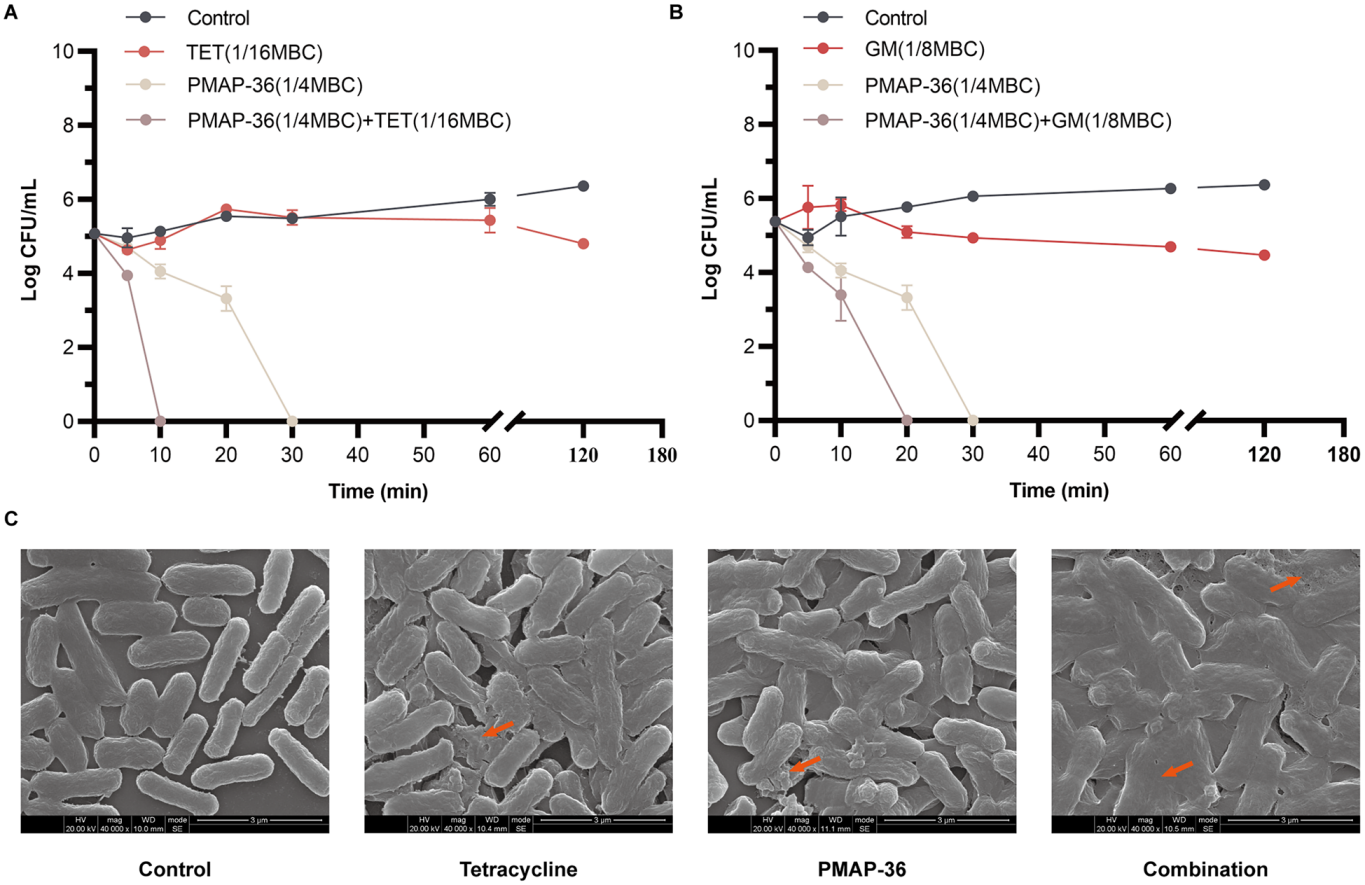
**Antibacterial efficacy of PMAP-36 in combination with antibiotics in vitro.** The time-kill curves of PMAP-36 in combination with tetracycline (TET) (**A**) and gentamicin (GM) (**B**) against PCN033 are shown. **C** SEM images of PCN033 after 4 h of treatment with tetracycline (1/16 MBC), PMAP-36 (1/4 MBC) or tetracycline (1/16 MBC) + PMAP-36 (1/4 MBC) are shown. Red arrows represent cell lysis.

The synergistic bactericidal effect of PMAP-36 in combination with tetracycline or gentamicin was further explored by a time-kill curve assay and SEM. As shown in Figures 2A, B, PMAP-36 and antibiotics (tetracycline and gentamicin) alone at sub-MBCs did not show obvious bactericidal activity against *E. coli* within 120 min, but PMAP-36 in combination with tetracycline or gentamicin at sub-MBCs killed all the bacteria within 30 min. In addition, PCN033 treated with tetracycline or PMAP-36 alone showed slight cell shrinkage under SEM, but combination treatment led to visible shrinkage of the cell wall and increased cell permeability and death (Figure 2C). These results indicate that PMAP-36 has strong synergistic bactericidal efficacy in combination with tetracycline in vitro.

### The effect of the combination of PMAP-36 and tetracycline on drug resistance in PCN033

**Figure 3:**
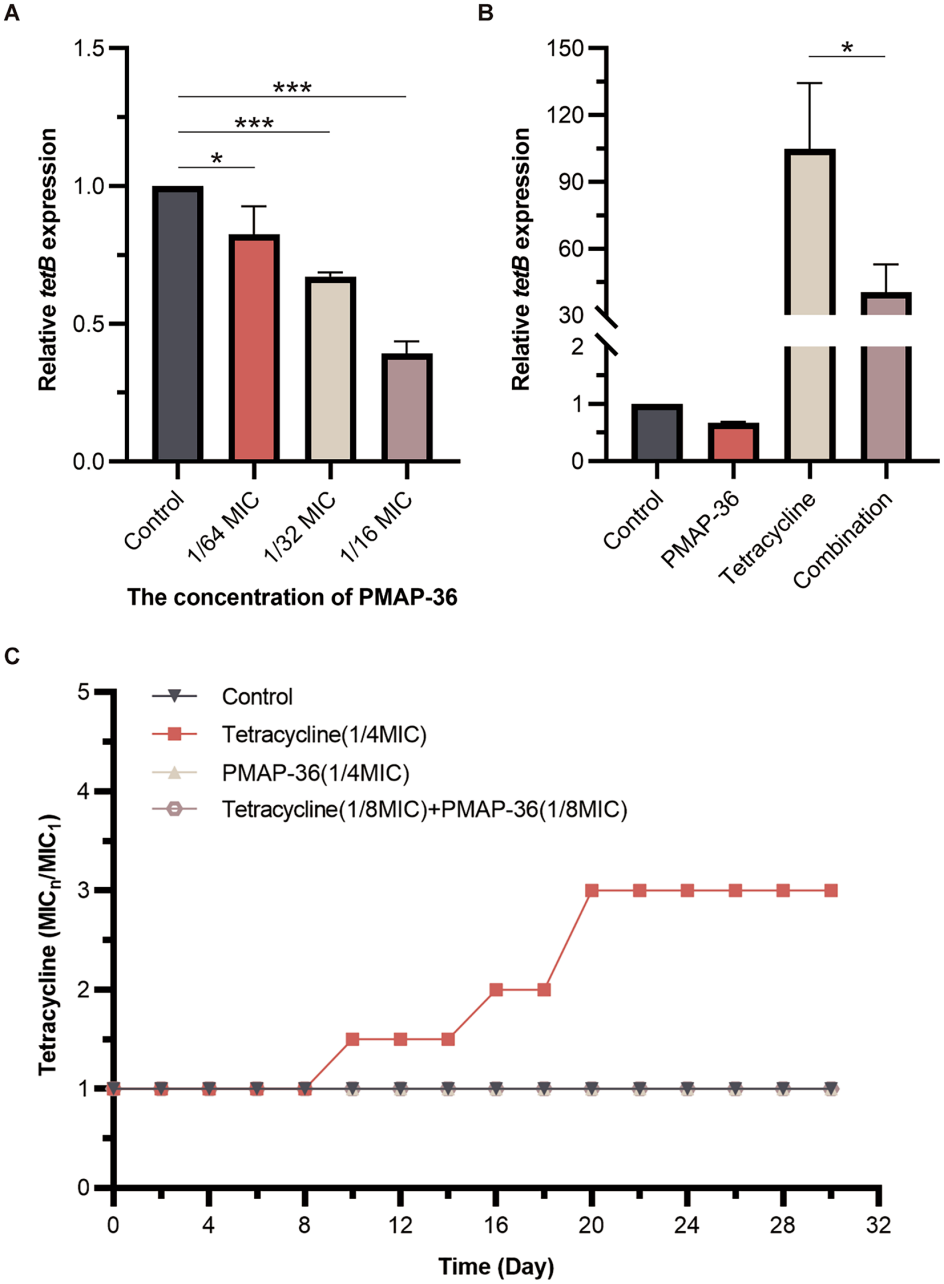
**The effect of the combination of PMAP-36 and tetracycline on drug resistance in PCN033.** PCN033 (1 × 10^6^ CFU) was incubated with different concentrations of PMAP-36, tetracycline (1/32 MIC), or tetracycline (1/32 MIC) + PMAP-36 (1/32 MIC) for 6 h at 37 °C, after which the mRNA levels of *tetB* were analysed by qPCR (**A**, **B**). PCN033 was exposed to sub-MICs of PMAP-36 and tetracycline at 37 °C for 30 generations, and the change in MIC was determined by normalizing the MIC of the *n* generation to the MIC of the first generation (**C**). Statistical significance is indicated by *p < 0.05 and ***p < 0.001 (Student’s *t* test).

To explore the effect of the combination of PMAP-36 and tetracycline on the sensitivity of PCN033 to antibiotics, transcription of the *tetB* gene in PCN033 was quantified. The qRT‒PCR results showed that PMAP-36 significantly inhibited the transcription of the *tetB* gene in a concentration-dependent manner (Figure 3A) and downregulated tetracycline-induced *tetB* mRNA expression (Figure 3B). Next, to further investigate the effect of the combination of PMAP-36 and tetracycline on the induction of bacterial drug resistance, PCN033 was exposed to sub-MIC concentrations of PMAP-36 and tetracycline for 30 generations. As shown in Figure 3C, the MIC of tetracycline increased by 1.5-fold in the 10th generation. After 30 generations, the MIC of tetracycline increased threefold. In contrast, treatment with PCN033 at sub-MICs of PMAP-36 and the combination of PMAP-36 and tetracycline did not cause any change. Taken together, these results suggest that PMAP-36 might restore the sensitivity of *E. coli* to tetracycline by inhibiting the transcription of *tetB*.

### Synergistic anti-infective effect of PMAP-36 in combination with tetracycline against *E. coli* infection in vivo

**Figure 4:**
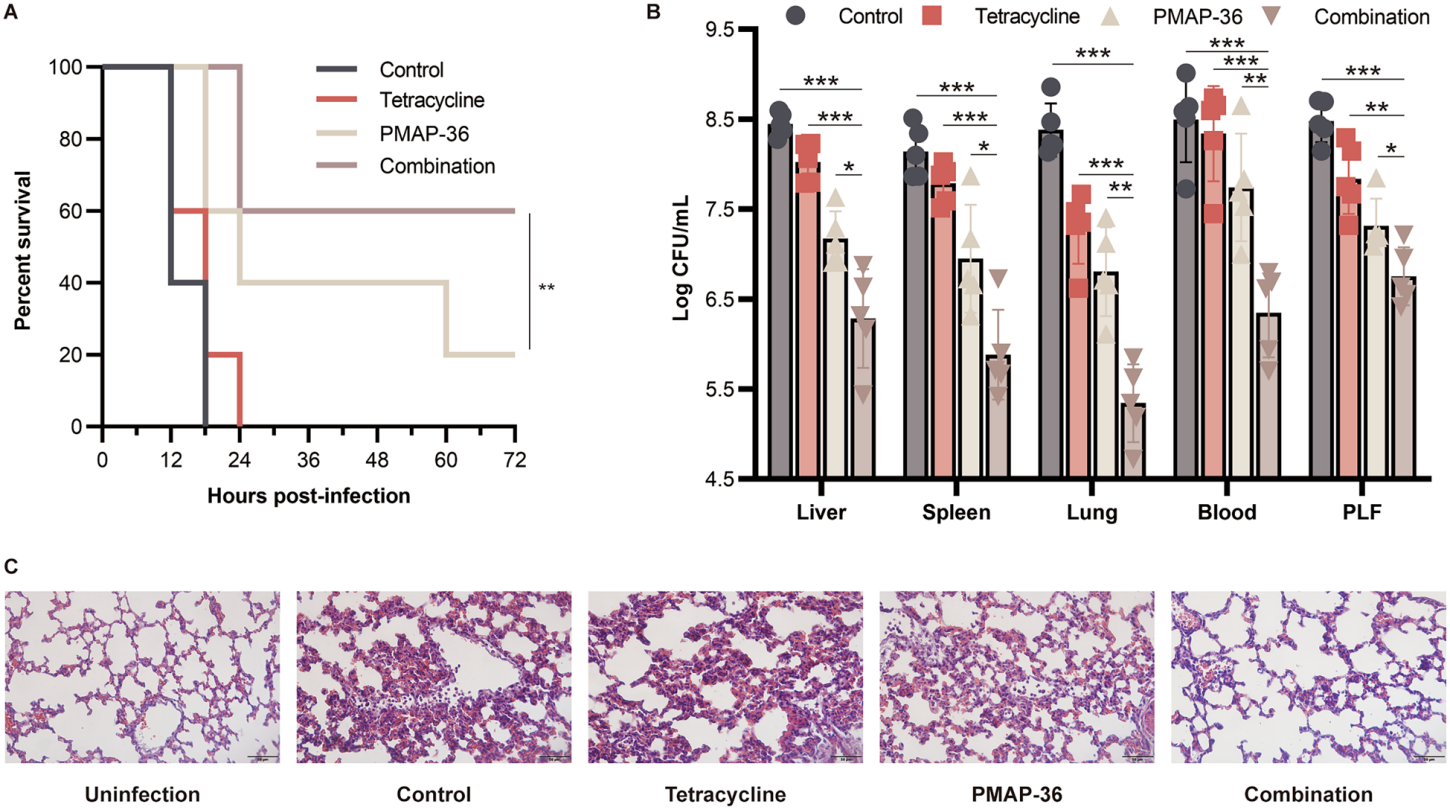
**Synergistic anti-infective effect of PMAP-36 in combination with tetracycline against *E. coli* infection in vivo.** Mice were intraperitoneally infected with PCN033 (1 × 10^7^ CFU) and then treated with tetracycline, PMAP-36, or PMAP-36 in combination with tetracycline. The infected mice treated with PBS were used as controls. The mice that survived at 72 h post-infection are shown (*n* = 10/group) (**A**). The bacterial burden in the liver, spleen, lung, blood, and peritoneal lavage fluid (PLF) was determined (*n* = 5/group) (**B**). Haematoxylin and eosin staining of the lung at 12 h post-infection was performed (original magnification × 400) (**C**). Images are representative of those of three mice from each group. Statistical significance is indicated by *p < 0.05, **p < 0.01, and ***p < 0.001 (Student’s *t* test).

**Figure 5:**
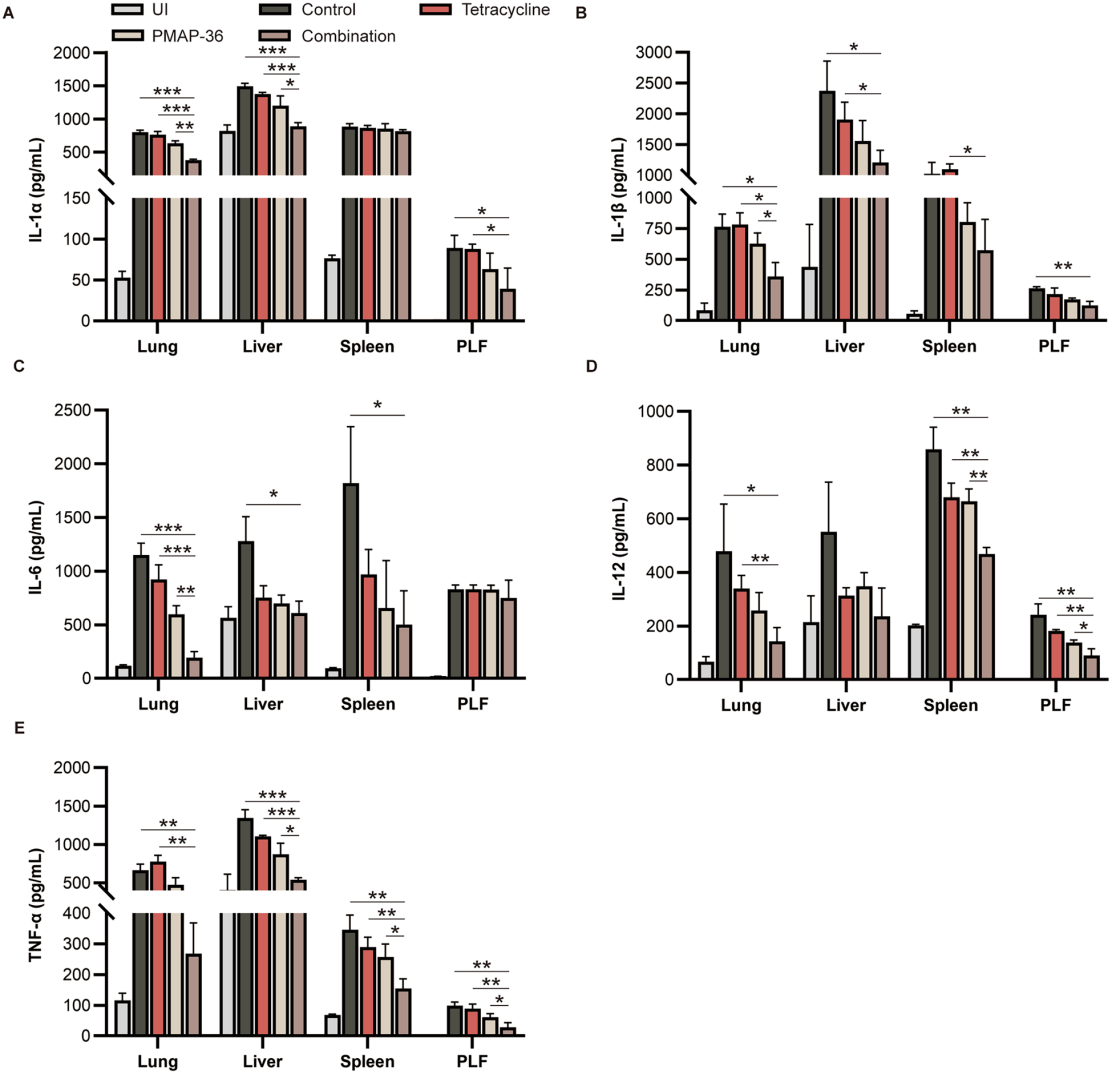
**The effect of PMAP-36 in combination with tetracycline on the production of inflammatory cytokines.** Mice were intraperitoneally infected with PCN033 (1 × 10^7^ CFU) and then treated with tetracycline, PMAP-36, or PMAP-36 in combination with tetracycline. The infected mice were treated with PBS as a control. At 12 h post-infection, the production of inflammatory cytokines, including IL-1α (**A**), IL-1β (**B**), IL-6 (**C**), IL-12 (**D**), and TNF-α (**E**), in the lung, liver, spleen and PLF was determined using ELISAs (*n* = 3/group). Statistical significance is indicated by *p < 0.05, **p < 0.01, and ***p < 0.001.

The synergistic effect of PMAP-36 in combination with tetracycline was further explored in a mouse infection model. As shown in Figure 4A, bacterial infection led to the death of all tetracycline-treated mice within 24 h, but treatment with the combination of PMAP-36 and tetracycline resulted in a relatively high survival rate (60%). Additionally, combination therapy significantly reduced the bacterial load in the liver, spleen, lung, blood and PLF compared with that in the nontreated mice and the tetracycline- or PMAP-36-treated mice (Figure 4B). Furthermore, the results of H&E staining showed that the lungs of untreated mice exhibited thickened alveolar walls and substantial inflammatory cell infiltration, but PMAP-36 treatment rescued the pathological changes in the lungs, and combination treatment with tetracycline significantly protected the lungs from infection (Figure 4C). Similarly, compared to the treatment of infected mice with or without tetracycline or PMAP-36 alone, the combination treatment of PMAP-36 and tetracycline significantly decreased the production of IL-1α (Figure 5A), IL-1β (Figure 5B), IL-6 (Figure 5C), IL-12 (Figure 5D) and TNF-α (Figure 5E) in the lung, liver, spleen, and PLF. Furthermore, PMAP-36 in combination with antibiotics (tetracycline or gentamicin) at sub-MBCs did not show significant cytotoxicity to macrophages (cell viability > 80%) or PK-15 cells (cell viability > 95%); as the combination also did not impact the haemolytic activity (< 5%) of erythrocytes (Additional file 1). These results indicate that the combination of PMAP-36 with tetracycline improves therapeutic efficacy in infected mice.

### PMAP-36 in combination with tetracycline promotes monocyte/macrophage recruitment

**Figure 6:**
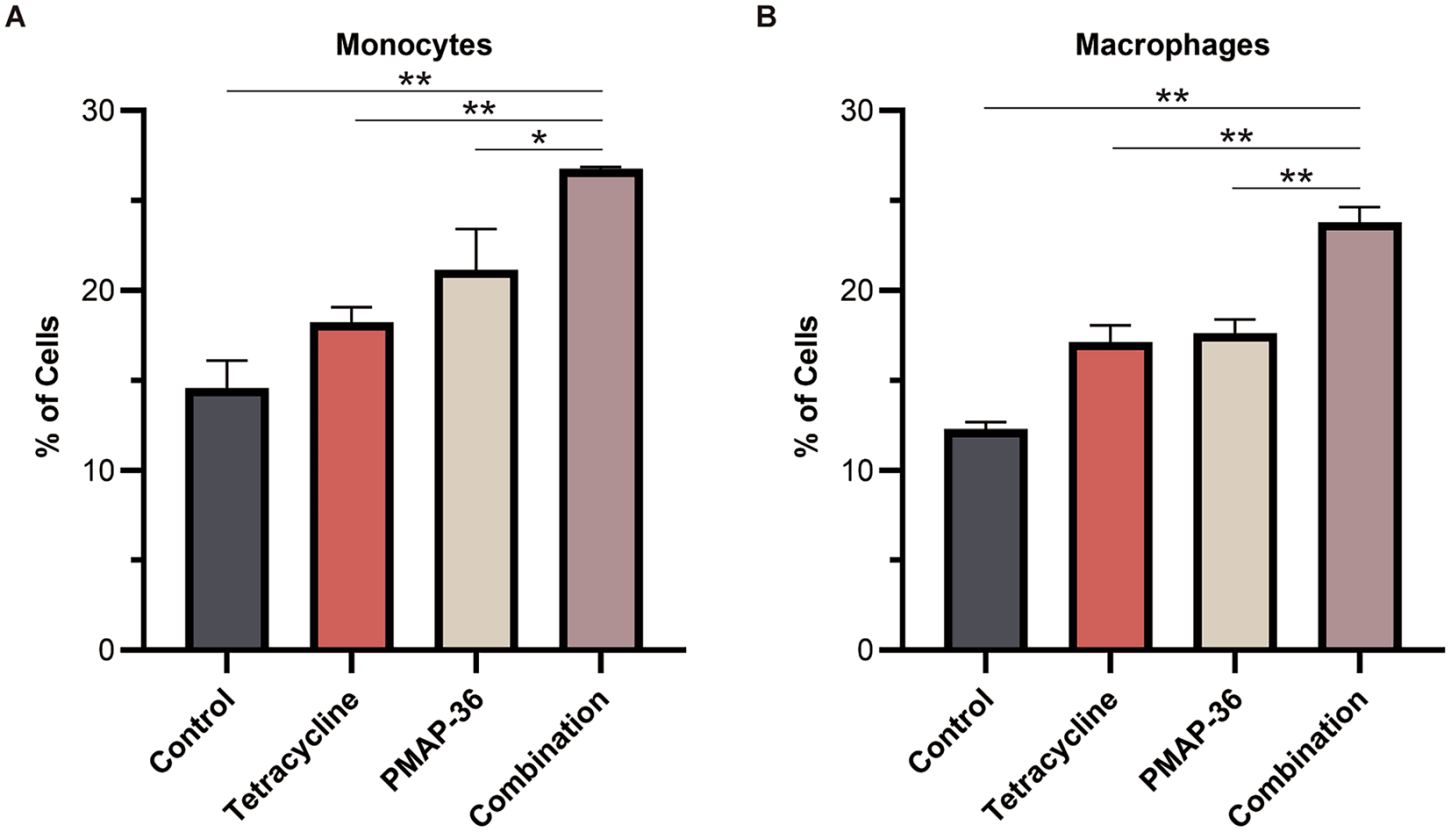
**PMAP-36 in combination with tetracycline promotes monocyte/macrophage recruitment.** Mice were intraperitoneally infected with PCN033 (1 × 10^7^ CFU) and then treated with PMAP-36 and tetracycline. Next, the PLFs were collected 12 h post-infection. The number of monocytes (**A**) and macrophages (**B**) were quantified by flow cytometry (*n* = 3/group). Statistical significance is indicated by *p < 0.05 and **p < 0.01.

Bacterial clearance in the host depends on the migration of phagocytes to sites of infection [17, 18]. To investigate whether PMAP-36 in combination with tetracycline promotes the recruitment of immune cells, the number of monocytes and macrophages in the PLF was determined. As shown in Figure 6A and B, treatment with PMAP-36 or tetracycline alone only slightly increased the number of monocytes and macrophages, but treatment with the combination of PMAP-36 and tetracycline significantly increased the recruitment of these cells, suggesting that the combination of PMAP-36 and tetracycline promotes the recruitment of phagocytes to the sites of infection [18]. Next, we investigated whether PMAP-36 combined with tetracycline promoted chemokine secretion to induce immune cell migration. The results showed that the combination of PMAP-36 and tetracycline did not affect production of the chemokines CXCL1 and CXCL2 in the PLF (Additional file 2).

## Discussion

Antimicrobial resistance is a major problem that not only threatens public health but also affects the safety of food animals [1]. Recently, ExPEC has been reported as an emerging problem in pig farming, where its incidence is increasing due to multidrug resistance, and ExPEC is causing severe infections of the central nervous system [19, 20]. Therefore, there is an urgent need to develop new antimicrobial agents to combat antibiotic resistance. AMPs have attracted extensive attention due to their broad spectrum of antimicrobial activity without the risk of inducing drug resistance [21]. Among the cathelicidin family of AMPs, PMAP-36 has a high net positive charge with 36% of its amino acids being cationic [22]. In addition to its high antimicrobial activity, PMAP-36 has multiple immunomodulatory activities. Previous studies have shown that PMAP-36 can neutralize LPS and inhibit LPS-induced macrophage activation; PMAP-36 also induces the expression of the chemokine CCL-2 [23–25]. The combination of conventional antibiotics with AMPs offers a productive strategy to reduce antibiotic resistance and hinder progression towards a “post-antibiotic” era [26]. Therefore, we investigated the synergistic bactericidal effect of PMAP-36 and tetracycline against porcine ExPEC in vitro and in vivo.

A variety of studies have reported the bactericidal activity of AMPs in combination with different conventional antibiotics. For example, the human AMP LL17-29 shows synergistic antimicrobial activity with chloramphenicol against methicillin-resistant *S. aureus* and multidrug-resistant *P. aeruginosa* [10]. In addition, nisin Z and pediocin synergize with several antibiotics, including penicillin, ampicillin, and rifampicin, to kill antibiotic-resistant *P. fluorescens* [27]. Similarly, we showed that the combination of PMAP-36 with tetracycline or gentamicin had strong bactericidal effects at sub-MBCs of peptides and antibiotics against multidrug-resistant porcine ExPEC. The antibacterial mechanism of most AMPs involves targeting the cell membrane [28]; therefore, they are unlikely to induce drug resistance. However, PMAP-36 induces the release of small vesicles at sub-MBCs in *E. coli,* as well as cell lysis and the clustering of DNA and ribosomes at the MBC, which have been previously shown to disrupt the inner membrane [23, 25]. Our study revealed no direct disruption of the cell membrane, but cell lysis was observed via SEM after treatment with PMAP-36 and tetracycline, indicating that PMAP-36-induced disruption of the cell membrane might promote the uptake of tetracycline, thereby affecting the synthesis of DNA and ribosomes. However, the exact antibacterial mechanism of PMAP-36 and tetracycline needs to be further studied. Notably, our study showed that the MBCs of PMAP-36 and tetracycline decreased fourfold and 16-fold, respectively, when used in combination, thereby delaying the evolution of bacterial drug resistance.

PCN033 harboured three plasmids, of which the largest plasmid (PCN033p3) contained the tetracycline gene (*tetB*), which is the main factor driving PCN033 resistance to tetracycline [13]. A recent study reported that the natural compound plumbagin in combination with tetracycline has a synergistic bactericidal effect against *E. coli* by inhibiting the expression of tetracycline resistance genes [29]. Wang et al. demonstrated that the AMP nisin in combination with oxacillin significantly inhibited the expression of β-lactam resistance gene (*mecA*) and recovered the sensitivity of methicillin-resistant *Staphylococcus aureus* to oxacillin [30]. In our study, we also found that the combination of PMAP-36 with tetracycline significantly reduced *tetB* transcription and even delayed the development of drug resistance. Therefore, we speculate that the synergistic bactericidal effect of PMAP-36 in combination with tetracycline occurs through the inhibition of *tetB* transcription.

Our in vivo study showed that PMAP-36 in combination with tetracycline significantly improved the mouse survival rate after *E. coli* challenge. A recent study reported that PMAP-36 and its analogues have antibacterial and anti-inflammatory effects against *Listeria monocytogenes* and *Salmonella choleraesuis* infections in vivo [31]. These studies demonstrated that PMAP-36 has effective therapeutic effects in vivo. Moreover, in our study, we showed that treatment with PMAP-36 alone or in combination with tetracycline reduced the bacterial load. Notably, PMAP-36 reportedly modulates the host immune response. For instance, PMAP-36 induces the production of the chemokine CCL-2 [25] and decreases cytokine expression in *Bordetella bronchiseptica*-derived outer membrane vesicle-stimulated macrophages [32]. Furthermore, AMPs reportedly exert chemotactic effects on leukocytes. For example, Marques-Neto et al. reported that the scorpion-derived AMP ToAP2A increases the recruitment of peritoneal macrophages and promotes the chemotactic migration of neutrophils [33]. In this study, treatment with a combination of PMAP-36 and tetracycline after porcine ExPEC infection in mice promoted the recruitment of monocytes and macrophages to the abdominal cavity, which might have resulted in the reduced bacterial load. In addition, the combination treatment significantly reduced the inflammatory response, decreased the production of inflammatory cytokines in the lung, spleen, liver and peritoneal lavage fluid, and alleviated pathological changes in the lung. However, the anti-inflammatory response induced by PMAP-36 and tetracycline was partially mediated by direct killing. Therefore, the exact anti-inflammatory mechanism of PMAP-36 still needs to be further studied.

In conclusion, PMAP-36 in combination with tetracycline had synergistic anti-infective effects on ExPEC both in vitro and in vivo. Importantly, the combination of PMAP-36 with tetracycline hinders the development of bacterial resistance. Furthermore, their combination inhibited the ExPEC-induced inflammatory response. Our study provides valuable information for the development of PMAP-36 as an agent that can be used with antibiotics for the treatment of bacterial infections.

### Supplementary Information

**Additional file 1**: **The cytotoxicity and haemolytic activity of PMAP-36 in combination with tetracycline or gentamicin.** The viability of peritoneal macrophages (A) and PK-15 cells (B) treated with PMAP-36 combined with tetracycline or gentamicin was determined via the WST-1 assay. The haemolytic activity of PMAP-36 in combination with tetracycline or gentamicin was determined using mouse erythrocytes (C).

**Additional file 2: PMAP-36 in combination with tetracycline does not affect the production of chemokines.** Mice were uninfected (UI) or intraperitoneally infected with PCN033 (1 × 10^7^ CFU) and then treated with PMAP-36 and tetracycline. PLFs were collected at 12 h post-infection, and the production of the chemokines CXCL1 (A) and CXCL2 (B) was measured by ELISA (*n* = 3/group).

## Data Availability

The data supporting the conclusions of this article are included within the article. Additional data used and/or analysed during the current study are available from the corresponding author upon reasonable request.
