# Supplementary figures and images for: Antibacterial activity of the antimicrobial peptide PMAP-36 in combination with tetracycline against porcine extraintestinal pathogenic Escherichia coli in vitro and in vivo
Source: Vet Res. 2024 Mar 22;55:35. doi: 10.1186/s13567-024-01295-w (PMC10960472; doi:10.1186/s13567-024-01295-w)

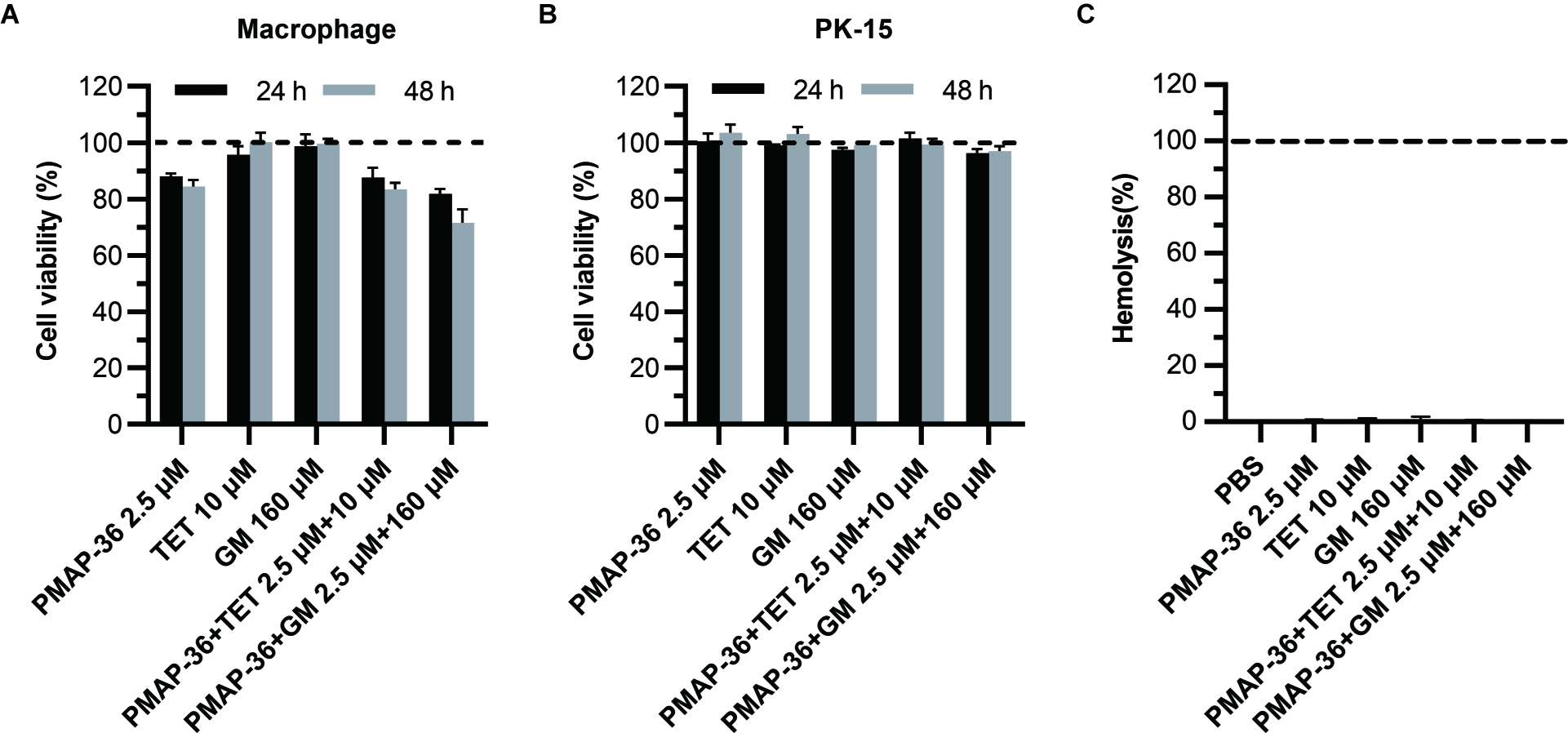

Supplement: Supplementary file 1 — Additional file 1: The cytotoxicity and haemolytic activity of PMAP-36 in combination with tetracycline or gentamicin. The viability of peritoneal macrophages (A) and PK-15 cells (B) treated with PMAP-36 combined with tetracycline or gentamicin was determined via the WST-1 assay. The haemolytic activity of PMAP-36 in combination with tetracycline or gentamicin was determined using mouse erythrocytes (C). [file 13567_2024_1295_MOESM1_ESM.tif]

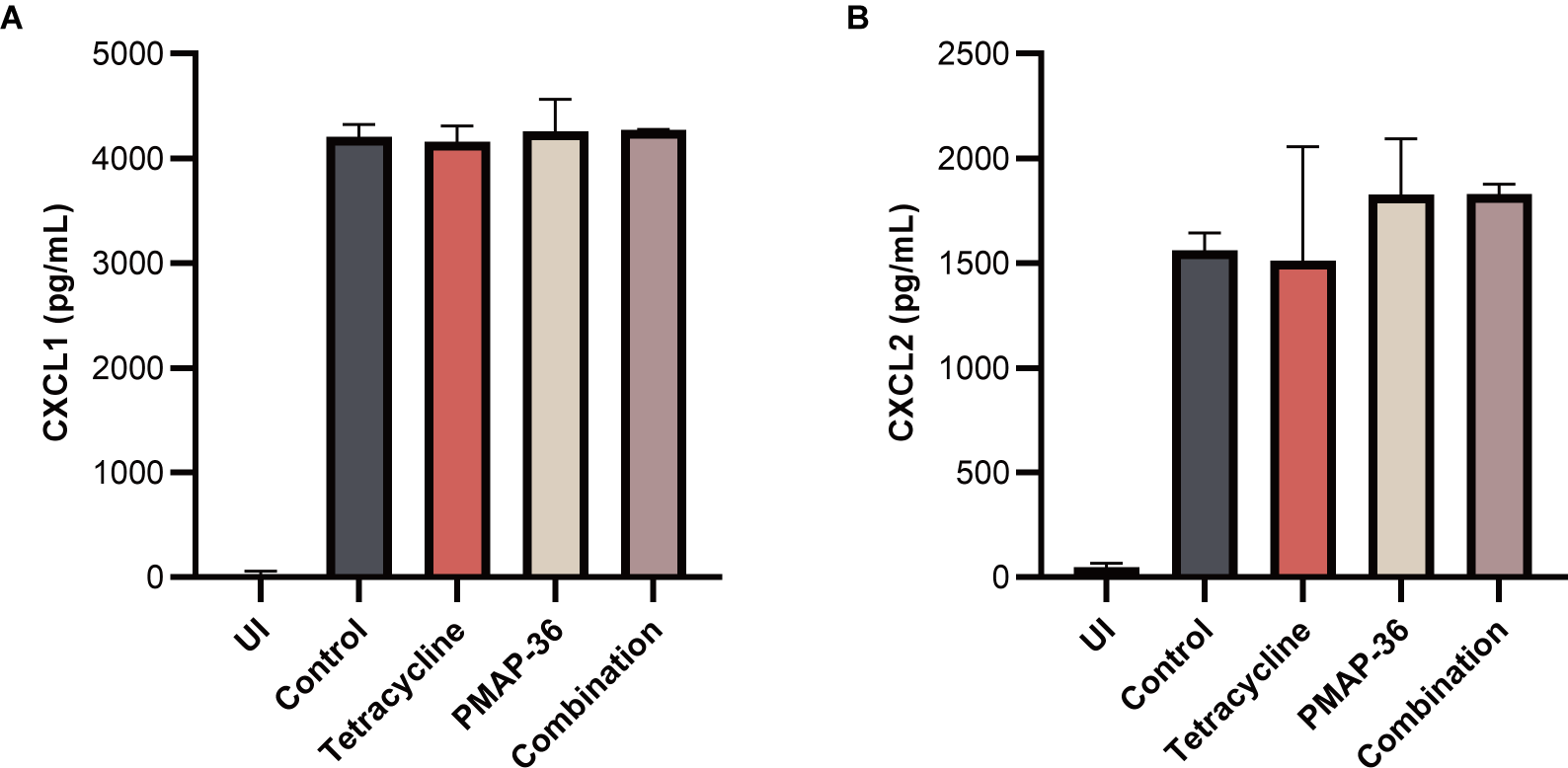

Supplement: Supplementary file 2 — Additional file 2: PMAP-36 in combination with tetracycline does not affect the production of chemokines. Mice were uninfected (UI) or intraperitoneally infected with PCN033 (1 × 10^7^ CFU) and then treated with PMAP-36 and tetracycline. PLFs were collected at 12 h post-infection, and the production of the chemokines CXCL1 (A) and CXCL2 (B) was measured by ELISA (n = 3/group). [file 13567_2024_1295_MOESM2_ESM.tif]
